# Crystal structure of *N*,*N*,*N*′,*N*′,*N*′′,*N*′′-hexa­methyl­guanidinium cyanate 1.5-hydrate

**DOI:** 10.1107/S2056989015024317

**Published:** 2015-12-24

**Authors:** Ioannis Tiritiris, Willi Kantlehner

**Affiliations:** a Fakultät Chemie/Organische Chemie, Hochschule Aalen, Beethovenstrasse 1, D-73430 Aalen, Germany

**Keywords:** crystal structure, cyanate, hexa­methyl­guanidinium, salt, O—H⋯O hydrogen bonds, O—H⋯N hydrogen bonds

## Abstract

The title hydrated salt, C_7_H_18_N_3_^+^·OCN^−^.1.5H_2_O, was synthesized starting from *N*,*N*,*N*′,*N*′,*N*′′,*N*′′-hexa­methyl­guanidinium chloride by a twofold anion-exchange reaction. The asymmetric unit contains two cations, two cyanate anions and three water mol­ecules. One cation shows orientational disorder and two sets of N-atom positions were found related by a 60° rotation, with an occupancy ratio of 0.852 (6):0.148 (6). The C—N bond lengths in both guanidin­ium ions range from 1.329 (2) to 1.358 (10) Å, indicating double-bond character, pointing towards charge delocalization within the NCN planes. Strong O—H⋯N hydrogen bonds between the crystal water mol­ecules and the cyanate ions and strong O—H⋯O hydrogen bonds between the water mol­ecules are present, resulting in a two-dimensional hydrogen bonded network running parallel to the (001) plane. The hexa­methyl­guanidinium ions are packed in between the layers built up by water mol­ecules and cyanate ions.

## Related literature   

For the synthesis of hexa­substituted guanidinium salts with different anions, see: Kantlehner *et al.* (1984 ▸). For the crystal structure of *N*,*N*,*N*′,*N*′,*N*′′,*N*′′-hexa­methyl­guanidinium chloride, see: Oelkers & Sundermeyer (2011 ▸). For the crystal structure of *N*,*N*,*N*′,*N*′,*N*′′,*N*′′-hexa­methyl­guanidinium di­fluoro­tri­methyl­silicate, see: Röschenthaler *et al.* (2002 ▸). For the crystal structure of *N*,*N*,*N*′,*N*′,*N*′′,*N*′′-hexa­methyl­guanidinium tetra­phenyl­borate, see: Frey *et al.* (1998 ▸). For the crystal structure of *N*,*N*,*N*′,*N*′,*N*′′,*N*′′-hexa­methyl­guanidinium fluoride, see: Kolomeitsev *et al.* (2000 ▸). For the crystal structure of *N*,*N*,*N*′,*N*′,*N*′′,*N*′′-hexa­methyl­guanidinium hexa­fluoro­silicate hexa­hydrate, see: Zhang *et al.* (1999 ▸). For the crystal structures of [C(NMe_2_)_3_][Mn(CO)_5_] and [C(NMe_2_)_3_][Co(CO)_4_], see: Petz & Weller (1991 ▸). For a neutron diffraction studie of deuterated ammonium cyanate, see: MacLean *et al.* (2003 ▸). For the use of intensity quotients and differences in absolute structure refinement, see: Parsons *et al.* (2013 ▸).
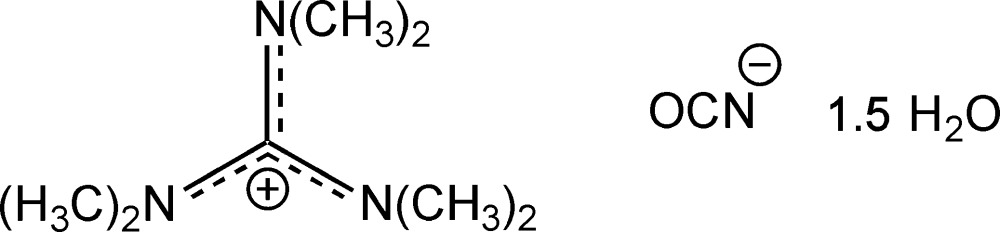


## Experimental   

### Crystal data  

2C_7_H_18_N_3_^+^·2CNO^−^·3H_2_O
*M* *_r_* = 426.58
Monoclinic, 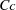

*a* = 8.3245 (5) Å
*b* = 22.536 (2) Å
*c* = 13.2580 (12) Å
β = 108.092 (7)°
*V* = 2364.2 (3) Å^3^
*Z* = 4
Mo *K*α radiation
μ = 0.09 mm^−1^
*T* = 100 K
0.40 × 0.25 × 0.10 mm

### Data collection  

Bruker Kappa APEXII DUO diffractometer
19766 measured reflections
5588 independent reflections
5279 reflections with *I* > 2σ(*I*)
*R*_int_ = 0.025
Standard reflections: 0

### Refinement  

*R*[*F* ^2^ > 2σ(*F* ^2^)] = 0.034
*wR*(*F* ^2^) = 0.089
*S* = 1.02
5588 reflections
328 parameters
2 restraints
H atoms treated by a mixture of independent and constrained refinement
Δρ_max_ = 0.40 e Å^−3^
Δρ_min_ = −0.19 e Å^−3^

### 

Data collection: *APEX2* (Bruker, 2008 ▸); cell refinement: *SAINT* (Bruker, 2008 ▸); data reduction: *SAINT*; program(s) used to solve structure: *SHELXS97* (Sheldrick, 2008 ▸); program(s) used to refine structure: *SHELXL2014* (Sheldrick, 2015 ▸); molecular graphics: *DIAMOND* (Brandenburg & Putz, 2005 ▸); software used to prepare material for publication: *SHELXL2014*.

## Supplementary Material

Crystal structure: contains datablock(s) I, global. DOI: 10.1107/S2056989015024317/rz5180sup1.cif

Structure factors: contains datablock(s) I. DOI: 10.1107/S2056989015024317/rz5180Isup2.hkl

**Fig. 1.**
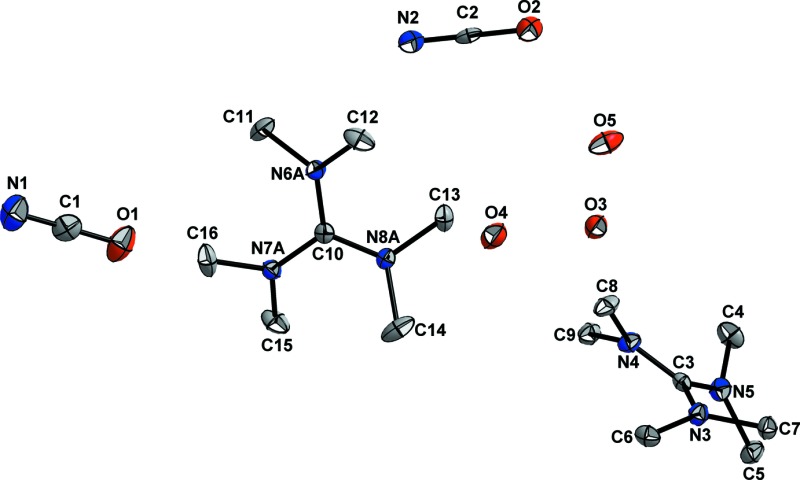
The structure of the title compound with displacement ellipsoids at the 50% probability level. All hydrogen atoms are omitted for clarity. Only the major component of the disordered cation is shown.

. DOI: 10.1107/S2056989015024317/rz5180fig1.tif
The structure of the title compound with displacement ellipsoids at the 50% probability level. All hydrogen atoms are omitted for clarity. Only the major component of the disordered cation is shown.

**Fig. 2.**
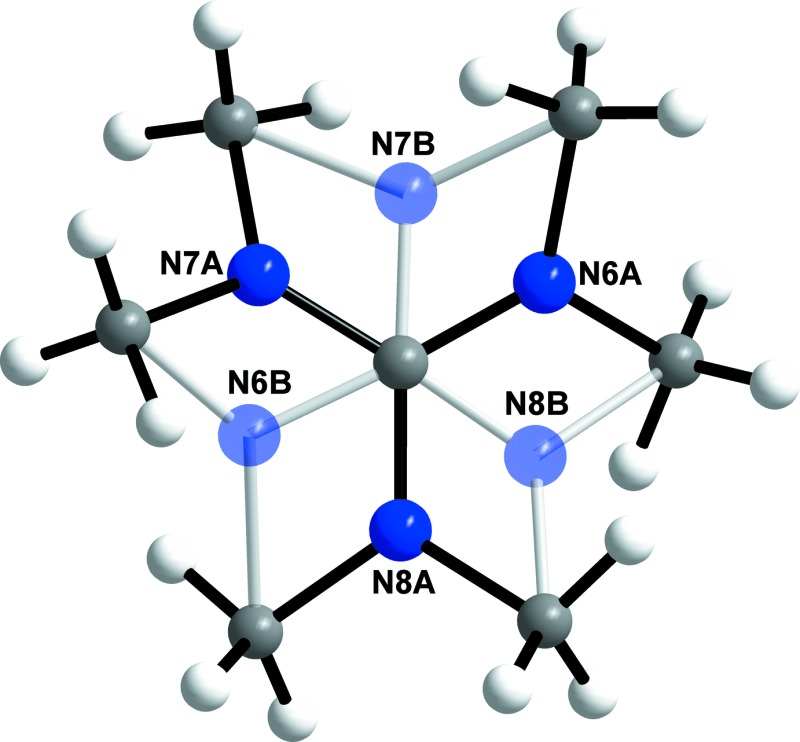
The structure of the orientationally disordered cation. The nitrogen atoms are disordered between the opaque and dark positions.

**Fig. 3.**
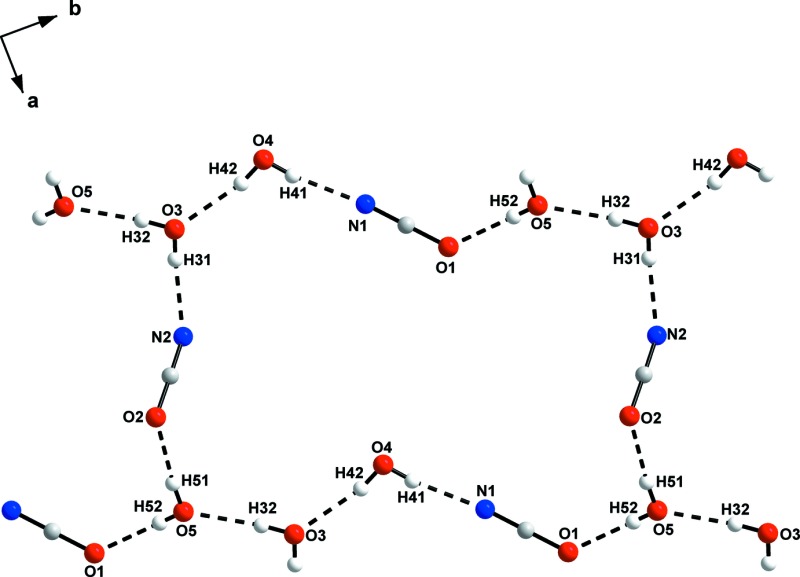
O—H···N and O—H···O hydrogen bonds (black dashed lines) between anions and water molecules and between the water molecules (view down the c axis).

**Fig. 4.**
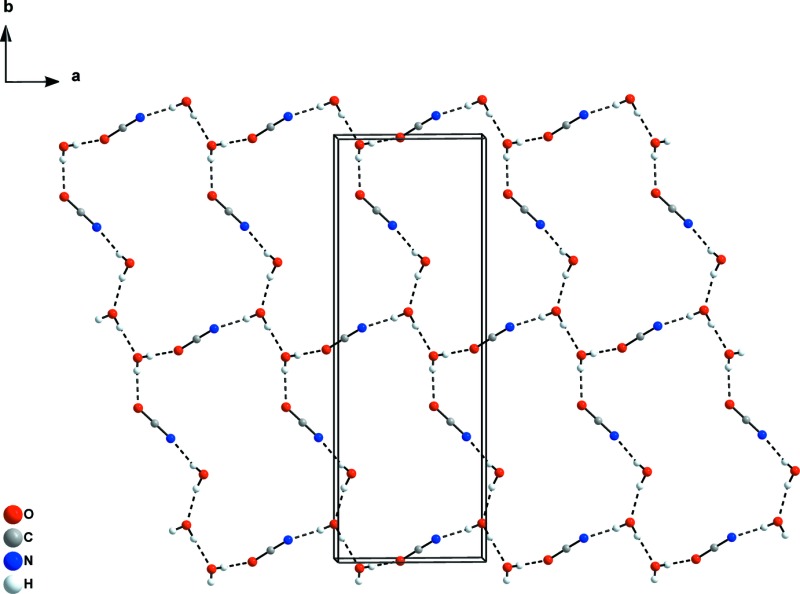
View down the c axis of the two-dimensional O—H···N and O—H···O hydrogen-bonding network (all hydrogen bonds are indicated by black dashed lines).

**Fig. 5.**
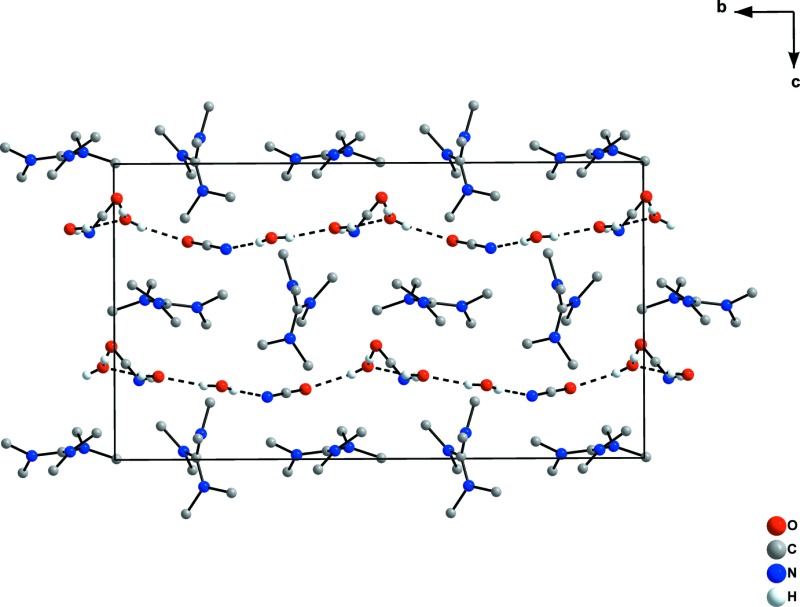
Packing of the guanidinium ions in between the layers build up by water molecules and cyanate ions (down the a axis).

. DOI: 10.1107/S2056989015024317/rz5180fig2.tif
The structure of the orientationally disordered cation. The nitro­gen atoms are disordered between the opaque and dark positions.

c . DOI: 10.1107/S2056989015024317/rz5180fig3.tif
O—H⋯N and O—H⋯O hydrogen bonds (black dashed lines) between anions and water mol­ecules and between the water mol­ecules (view down the *c* axis).

c . DOI: 10.1107/S2056989015024317/rz5180fig4.tif
View down the *c* axis of the two-dimensional O—H⋯N and O—H⋯O hydrogen-bonding network (all hydrogen bonds are indicated by black dashed lines).

a . DOI: 10.1107/S2056989015024317/rz5180fig5.tif
Packing of the guanidinium ions in between the layers build up by water mol­ecules and cyanate ions (down the *a* axis).

CCDC reference: 867308

Additional supporting information:  crystallographic information; 3D view; checkCIF report

## Figures and Tables

**Table 1:** Hydrogen-bond geometry (Å, °)

*D*—H⋯*A*	*D*—H	H⋯*A*	*D*⋯*A*	*D*—H⋯*A*
O3—H31⋯N2	0.78 (3)	2.00 (3)	2.780 (3)	176 (3)
O3—H32⋯O5^i^	0.86 (4)	2.00 (4)	2.858 (4)	172 (3)
O4—H42⋯O3^ii^	0.83 (4)	2.04 (4)	2.852 (4)	164 (3)
O4—H41⋯N1^ii^	0.84 (3)	2.00 (3)	2.833 (3)	173 (3)
O5—H51⋯O2^iii^	0.85 (3)	1.92 (3)	2.761 (3)	175 (3)
O5—H52⋯O1^iv^	0.74 (4)	2.10 (4)	2.840 (4)	177 (3)

Symmetry codes: (i) 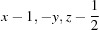; (ii) 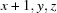; (iii) 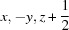; (iv) 
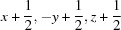
.

## supplementary crystallographic information

### S1. Comment

The reaction of phosgene with *N*,*N*,*N*',*N*'-tetramethylurea yields *N*,*N*,*N*',*N*'-tetramethylchloroformamidinium chloride, which can be transformed by a mixture of dimethylamine and triethylamine into a mixture of *N*,*N*,*N*',*N*',*N*'',*N*''-hexamethylguanidinium chloride and triethylamine hydrochloride. Treating the salt mixture with an aqueous sodium hydroxide solution leads after work up to the pure guanidinium chloride. Conversion of the chloride to the tetrafluoroborate salt occurs by heating it with BF_3_O(C_2_H_5_)_2_ (Kantlehner *et al.*, 1984). A further anion exchange was possible by reacting *N*,*N*,*N*',*N*',*N*'',*N*''-hexamethylguanidinium tetrafluoroborate with potassium cyanate in water. According to the structure analysis of the title compound, the asymmetric unit contains two *N*,*N*,*N*',*N*',*N*'',*N*''-hexamethylguanidinium (HMG^+^) ions, two cyanate ions and three water molecules (Fig. 1). One cation (cation I) shows an orientational disorder and two sets of N positions were found related by a 60° rotation, with an occupancy ratio of 0.852 (6):0.148 (6). This leads to the characteristic star-shaped appearance of the HMG^+^ ion (Fig. 2). The second cation (cation II) is not disordered. Searching for known crystal structures in literature of *N*,*N*,*N*',*N*',*N*'',*N*''-hexamethylguanidinium salts [see, for example: chloride salt (Oelkers & Sundermeyer, 2011), difluorotrimethylsilicate salt (Röschenthaler *et al.*, 2002), tetraphenylborate salt (Frey *et al.*, 1998), fluoride salt (Kolomeitsev *et al.*, 2000), hexafluorosilicate hexahydrate salt (Zhang *et al.*, 1999), [Mn(CO)_5_] and [Co(CO)_4_] salts (Petz & Weller, 1991)], it is obvious that in all those compounds the HMG^+^ ions are orientationally disordered too. In the title salt, the C–N bond lengths of both cations are in a range from 1.329 (2) and 1.358 (10) Å, indicating double bond character. The CN_3_ units are planar and the N–C–N angles are ranging from 118.0 (7)° to 121.8 (7)°. The positive charge is completely delocalized in the CN_3_ plane. The N–C bond lengths in the non-disordered guanidinium ion (cation II) are in a typical range from 1.453 (3) to 1.475 (2) Å, characteristic for a N–C single bond. In the disordered one (cation I), some N–C bond lengths deviate from their typical values and appear to be slightly longer [*d*(N–C) = 1.464 (3) − 1.655 (10) Å]. The N–C and C–O bond lengths in both cyanate ions [*d*(N–C) = 1.165 (3) and 1.172 (3) Å; d(C–O) = 1.213 (3) and 1.230 (3) Å] are in very good agreement with the data determined from a neutron diffraction study of deuterated ammonium cyanate (ND_4_OCN) at 14 K [*d*(N–C) = 1.191 (5) Å and d(C–O) =) 1.215 (5) Å (MacLean *et al.*, 2003)]. Strong O–H···N hydrogen bonds between the crystal water molecules and the cyanate ions [*d*(H···N) = 2.00 (3) Å (Tab. 1)] and strong O–H···O hydrogen bonds between the water molecules are present [*d*(H···O) = 1.92 (3) − 2.10 (4) Å (Tab. 1)] (Fig. 3), resulting in a two-dimensional hydrogen bonded network parallel to the (0 0 1) plane (Fig. 4). Additionally, C–H···N and C–H···O interactions between the H atoms of the guanidinium –N(CH_3_)_2_ groups and the cyanate ions are present [*d*(H···N) = 2.52 – 2.61 Å; d(H···O) = 2.46 – 2.60 Å]. The hexamethylguanidinium ions are packed in between the layers build up by water molecules and cyanate ions (Fig. 5).

### S2. Experimental

To a solution of 10 g (0.043 mol) *N*,*N*,*N*',*N*',*N*'',*N*''-hexamethylguanidinium tetrafluoroborate in water, 3.51 g (0.043 mol) potassium cyanate in 20 ml water was added. The solution was kept for twelve hours at 273 K and the precipitated potassium tetrafluoroborate was removed by filtration. After removing of the water, the residue was redissolved in acetonitrile and the solution was filtered again to remove the insoluble residue. The title compound crystallized from a saturated acetonitrile solution after several days at 273 K, forming colorless single crystals. Yield: 7.05 g (88%).

### S3. Refinement

The O-bound H atoms of the water molecules were located in a difference Fourier map and were refined freely [O—H = 0.74 (4) − 0.86 (4) Å]. The atoms N6, N7 and N8 of one cation are disordered over two sets of sites (N6A, N7A and N8A; N6B, N7B and N8B) with refined occupancies of 0.862 (6):0.138 (6), 0.852 (6):0.148 (6) and 0.852 (6):0.148 (6). The title compound crystallizes in the non-centrosymmetric space group *Cc*; however, in the absence of significant anomalous scattering effects, the determined Flack parameter *x* = 0.2 (3) (Parsons *et al.*, 2013) is essentially meaningless. The hydrogen atoms of the methyl groups were allowed to rotate with a fixed angle around the C–N bonds to best fit the experimental electron density, with *U*_iso_(H) set to 1.5 *U*_eq_(C) and d(C—H) = 0.98 Å.

### Crystal data

**Table:**

2C_7_H_18_N_3_^+^·2CNO^−^·3H_2_O	*F*(000) = 936
*M**_r_* = 426.58	*D*_x_ = 1.199 Mg m^−^^3^
Monoclinic, *C**c*	Mo *K*α radiation, λ = 0.71073 Å
*a* = 8.3245 (5) Å	Cell parameters from 19766 reflections
*b* = 22.536 (2) Å	θ = 1.8–28.4°
*c* = 13.2580 (12) Å	µ = 0.09 mm^−^^1^
β = 108.092 (7)°	*T* = 100 K
*V* = 2364.2 (3) Å^3^	Block, colorless
*Z* = 4	0.40 × 0.25 × 0.10 mm

### Data collection

**Table:**

Bruker Kappa APEXII DUO diffractometer	5279 reflections with *I* > 2σ(*I*)
Radiation source: fine-focus sealed tube	*R*_int_ = 0.025
Triumph monochromator	θ_max_ = 28.4°, θ_min_ = 1.8°
φ scans, and ω scans	*h* = −11→11
19766 measured reflections	*k* = −29→30
5588 independent reflections	*l* = −17→17

### Refinement

**Table:**

Refinement on *F*^2^	Primary atom site location: structure-invariant direct methods
Least-squares matrix: full	Secondary atom site location: difference Fourier map
*R*[*F*^2^ > 2σ(*F*^2^)] = 0.034	Hydrogen site location: inferred from neighbouring sites
*wR*(*F*^2^) = 0.089	H atoms treated by a mixture of independent and constrained refinement
*S* = 1.02	*w* = 1/[σ^2^(*F*_o_^2^) + (0.0493*P*)^2^ + 0.9721*P*] where *P* = (*F*_o_^2^ + 2*F*_c_^2^)/3
5588 reflections	(Δ/σ)_max_ < 0.001
328 parameters	Δρ_max_ = 0.40 e Å^−^^3^
2 restraints	Δρ_min_ = −0.19 e Å^−^^3^

### Special details

**Table:**

Geometry. All e.s.d.'s (except the e.s.d. in the dihedral angle between two l.s. planes) are estimated using the full covariance matrix. The cell e.s.d.'s are taken into account individually in the estimation of e.s.d.'s in distances, angles and torsion angles; correlations between e.s.d.'s in cell parameters are only used when they are defined by crystal symmetry. An approximate (isotropic) treatment of cell e.s.d.'s is used for estimating e.s.d.'s involving l.s. planes.
Refinement. Refinement of *F*^2^ against ALL reflections. The weighted *R*-factor *wR* and goodness of fit *S* are based on *F*^2^, conventional *R*-factors *R* are based on *F*, with *F* set to zero for negative *F*^2^. The threshold expression of *F*^2^ > σ(*F*^2^) is used only for calculating *R*-factors(gt) *etc*. and is not relevant to the choice of reflections for refinement. *R*-factors based on *F*^2^ are statistically about twice as large as those based on *F*, and *R*- factors based on ALL data will be even larger.

### Fractional atomic coordinates and isotropic or equivalent isotropic displacement parameters (Å^2^)

**Table:**

	*x*	*y*	*z*	*U*_iso_*/*U*_eq_	Occ. (<1)
O1	0.3316 (2)	0.36058 (8)	0.23689 (17)	0.0409 (4)	
C1	0.2204 (3)	0.32390 (9)	0.22404 (19)	0.0269 (4)	
N1	0.1126 (3)	0.28850 (9)	0.2104 (2)	0.0397 (5)	
C2	0.4331 (2)	0.02149 (8)	0.32420 (15)	0.0181 (4)	
O2	0.5548 (2)	−0.00567 (8)	0.38261 (13)	0.0316 (4)	
N2	0.3167 (2)	0.04653 (9)	0.26863 (17)	0.0300 (4)	
C3	0.1363 (2)	0.09910 (8)	0.52739 (14)	0.0150 (3)	
N3	0.2570 (2)	0.05814 (7)	0.54024 (13)	0.0194 (3)	
N4	−0.0178 (2)	0.08430 (7)	0.52842 (14)	0.0200 (3)	
N5	0.1730 (2)	0.15661 (7)	0.51504 (14)	0.0206 (3)	
C4	0.0955 (3)	0.20481 (9)	0.55702 (18)	0.0258 (4)	
H4A	0.0156	0.2262	0.4982	0.039*	
H4B	0.1835	0.2322	0.5974	0.039*	
H4C	0.0355	0.1885	0.6037	0.039*	
C5	0.2881 (3)	0.17368 (10)	0.45722 (18)	0.0245 (4)	
H5A	0.3885	0.1925	0.5060	0.037*	
H5B	0.2316	0.2017	0.4008	0.037*	
H5C	0.3221	0.1383	0.4258	0.037*	
C6	0.2163 (3)	−0.00338 (8)	0.50317 (16)	0.0208 (4)	
H6A	0.2274	−0.0294	0.5642	0.031*	
H6B	0.2943	−0.0165	0.4655	0.031*	
H6C	0.1001	−0.0052	0.4551	0.031*	
C7	0.4348 (2)	0.07080 (9)	0.59433 (16)	0.0198 (4)	
H7A	0.4983	0.0686	0.5433	0.030*	
H7B	0.4799	0.0416	0.6508	0.030*	
H7C	0.4458	0.1107	0.6251	0.030*	
C8	−0.0467 (3)	0.03409 (10)	0.59113 (17)	0.0243 (4)	
H8A	−0.0926	0.0005	0.5440	0.036*	
H8B	−0.1274	0.0458	0.6279	0.036*	
H8C	0.0604	0.0225	0.6434	0.036*	
C9	−0.1698 (2)	0.11617 (9)	0.46464 (17)	0.0216 (4)	
H9A	−0.2186	0.1382	0.5118	0.032*	
H9B	−0.2528	0.0876	0.4229	0.032*	
H9C	−0.1391	0.1439	0.4167	0.032*	
N6A	0.3149 (2)	0.16312 (8)	0.08657 (15)	0.0176 (5)	0.862 (6)
N7A	0.2872 (2)	0.16823 (8)	−0.09169 (15)	0.0176 (5)	0.852 (6)
N8A	0.5332 (2)	0.13023 (8)	0.02745 (15)	0.0165 (5)	0.852 (6)
N6B	0.4372 (13)	0.1485 (5)	−0.0765 (8)	0.011 (3)	0.138 (6)
N7B	0.2238 (14)	0.1787 (5)	−0.0095 (9)	0.017 (3)	0.148 (6)
N8B	0.4759 (12)	0.1399 (4)	0.1027 (7)	0.011 (3)	0.148 (6)
C10	0.3786 (2)	0.15393 (7)	0.00727 (15)	0.0140 (3)	
C11	0.1322 (3)	0.15698 (9)	0.0706 (2)	0.0257 (4)	
H11A	0.0761	0.1414	−0.0007	0.038*	
H11B	0.0846	0.1959	0.0784	0.038*	
H11C	0.1146	0.1295	0.1236	0.038*	
C12	0.4256 (3)	0.18015 (10)	0.19285 (17)	0.0286 (5)	
H12A	0.5401	0.1874	0.1900	0.043*	
H12B	0.4282	0.1480	0.2431	0.043*	
H12C	0.3818	0.2163	0.2160	0.043*	
C13	0.6009 (2)	0.08765 (9)	0.11404 (17)	0.0218 (4)	
H13A	0.5138	0.0783	0.1471	0.033*	
H13B	0.6992	0.1050	0.1672	0.033*	
H13C	0.6349	0.0513	0.0857	0.033*	
C14	0.6438 (3)	0.14659 (10)	−0.0358 (2)	0.0318 (5)	
H14A	0.5891	0.1775	−0.0868	0.048*	
H14B	0.6643	0.1116	−0.0740	0.048*	
H14C	0.7516	0.1615	0.0113	0.048*	
C15	0.2985 (3)	0.13442 (10)	−0.18353 (16)	0.0274 (4)	
H15A	0.3685	0.0991	−0.1591	0.041*	
H15B	0.3495	0.1592	−0.2261	0.041*	
H15C	0.1849	0.1223	−0.2268	0.041*	
C16	0.1731 (3)	0.22027 (9)	−0.1124 (2)	0.0296 (5)	
H16A	0.1823	0.2404	−0.0453	0.044*	
H16B	0.0563	0.2071	−0.1455	0.044*	
H16C	0.2051	0.2478	−0.1602	0.044*	
O3	0.0041 (2)	0.08290 (7)	0.28078 (13)	0.0251 (3)	
H31	0.090 (4)	0.0710 (12)	0.277 (2)	0.025 (7)*	
H32	−0.055 (4)	0.0523 (16)	0.285 (3)	0.044 (9)*	
O4	0.8891 (2)	0.20268 (8)	0.24797 (15)	0.0327 (4)	
H41	0.962 (4)	0.2257 (13)	0.237 (2)	0.027 (7)*	
H42	0.934 (5)	0.1695 (18)	0.250 (3)	0.054 (10)*	
O5	0.8322 (2)	0.02166 (9)	0.81332 (15)	0.0330 (4)	
H51	0.747 (4)	0.0190 (13)	0.835 (2)	0.031 (7)*	
H52	0.833 (4)	0.0529 (16)	0.795 (3)	0.039 (9)*	

### Atomic displacement parameters (Å^2^)

**Table:**

	*U*^11^	*U*^22^	*U*^33^	*U*^12^	*U*^13^	*U*^23^
O1	0.0293 (9)	0.0276 (8)	0.0665 (13)	−0.0008 (7)	0.0161 (9)	0.0045 (8)
C1	0.0280 (10)	0.0187 (9)	0.0352 (12)	0.0088 (8)	0.0114 (9)	0.0042 (8)
N1	0.0375 (11)	0.0197 (9)	0.0630 (15)	−0.0025 (8)	0.0172 (11)	−0.0015 (9)
C2	0.0195 (9)	0.0180 (8)	0.0223 (9)	−0.0058 (7)	0.0147 (7)	−0.0039 (7)
O2	0.0260 (8)	0.0351 (9)	0.0356 (9)	0.0022 (7)	0.0123 (7)	0.0047 (7)
N2	0.0277 (9)	0.0340 (10)	0.0329 (10)	0.0041 (8)	0.0162 (8)	0.0065 (8)
C3	0.0180 (8)	0.0153 (8)	0.0111 (8)	−0.0003 (6)	0.0038 (6)	−0.0008 (6)
N3	0.0186 (8)	0.0171 (7)	0.0222 (8)	−0.0009 (6)	0.0061 (6)	−0.0008 (6)
N4	0.0209 (8)	0.0191 (8)	0.0218 (8)	−0.0010 (6)	0.0090 (6)	0.0019 (6)
N5	0.0226 (8)	0.0166 (7)	0.0230 (8)	−0.0009 (6)	0.0080 (6)	0.0006 (6)
C4	0.0331 (11)	0.0153 (9)	0.0274 (10)	0.0048 (7)	0.0069 (8)	−0.0030 (8)
C5	0.0249 (10)	0.0244 (10)	0.0262 (10)	−0.0068 (8)	0.0108 (8)	0.0029 (8)
C6	0.0278 (10)	0.0142 (8)	0.0219 (9)	0.0022 (7)	0.0102 (7)	−0.0001 (7)
C7	0.0154 (8)	0.0209 (9)	0.0218 (9)	−0.0004 (7)	0.0039 (7)	−0.0006 (7)
C8	0.0252 (10)	0.0275 (10)	0.0247 (10)	−0.0029 (8)	0.0142 (8)	0.0060 (8)
C9	0.0178 (9)	0.0231 (9)	0.0246 (10)	0.0021 (7)	0.0073 (7)	−0.0004 (8)
N6A	0.0164 (10)	0.0201 (9)	0.0175 (10)	−0.0003 (7)	0.0068 (7)	−0.0014 (7)
N7A	0.0198 (9)	0.0179 (10)	0.0149 (9)	0.0020 (7)	0.0050 (7)	0.0007 (7)
N8A	0.0145 (9)	0.0168 (9)	0.0181 (9)	0.0002 (7)	0.0050 (7)	−0.0002 (7)
N6B	0.013 (5)	0.017 (6)	0.006 (5)	−0.001 (4)	0.006 (4)	−0.001 (4)
N7B	0.016 (5)	0.016 (5)	0.024 (6)	0.006 (4)	0.014 (4)	0.005 (4)
N8B	0.016 (5)	0.012 (5)	0.003 (4)	0.000 (4)	0.003 (3)	−0.001 (3)
C10	0.0148 (7)	0.0102 (7)	0.0163 (8)	−0.0019 (6)	0.0038 (6)	−0.0013 (6)
C11	0.0200 (9)	0.0248 (10)	0.0379 (12)	0.0004 (8)	0.0173 (9)	−0.0008 (9)
C12	0.0421 (13)	0.0267 (11)	0.0163 (9)	−0.0055 (9)	0.0084 (9)	−0.0055 (8)
C13	0.0167 (8)	0.0183 (9)	0.0267 (10)	0.0021 (7)	0.0011 (7)	0.0037 (8)
C14	0.0263 (11)	0.0293 (11)	0.0492 (14)	−0.0076 (9)	0.0251 (10)	−0.0115 (10)
C15	0.0394 (12)	0.0268 (10)	0.0144 (9)	−0.0007 (9)	0.0060 (8)	−0.0044 (8)
C16	0.0240 (10)	0.0211 (9)	0.0356 (12)	0.0016 (8)	−0.0026 (9)	0.0095 (9)
O3	0.0214 (7)	0.0235 (7)	0.0314 (8)	0.0013 (6)	0.0097 (6)	0.0028 (6)
O4	0.0283 (8)	0.0272 (8)	0.0483 (10)	0.0062 (7)	0.0200 (7)	0.0074 (7)
O5	0.0313 (9)	0.0332 (9)	0.0414 (10)	0.0117 (7)	0.0211 (8)	0.0132 (8)

### Geometric parameters (Å, º)

**Table:**

O1—C1	1.213 (3)	N8A—C10	1.341 (3)
C1—N1	1.172 (3)	N8A—C13	1.468 (3)
C2—N2	1.165 (3)	N8A—C14	1.472 (3)
C2—O2	1.230 (3)	N6B—C10	1.350 (10)
C3—N4	1.329 (2)	N6B—C15	1.558 (10)
C3—N3	1.336 (2)	N6B—C14	1.635 (11)
C3—N5	1.353 (2)	N7B—C10	1.358 (10)
N3—C7	1.459 (2)	N7B—C11	1.566 (11)
N3—C6	1.475 (2)	N7B—C16	1.600 (11)
N4—C8	1.468 (3)	N8B—C10	1.311 (9)
N4—C9	1.472 (3)	N8B—C13	1.548 (10)
N5—C5	1.453 (3)	N8B—C12	1.655 (10)
N5—C4	1.459 (3)	C11—H11A	0.9800
C4—H4A	0.9800	C11—H11B	0.9800
C4—H4B	0.9800	C11—H11C	0.9800
C4—H4C	0.9800	C12—H12A	0.9800
C5—H5A	0.9800	C12—H12B	0.9800
C5—H5B	0.9800	C12—H12C	0.9800
C5—H5C	0.9800	C13—H13A	0.9800
C6—H6A	0.9800	C13—H13B	0.9800
C6—H6B	0.9800	C13—H13C	0.9800
C6—H6C	0.9800	C14—H14A	0.9800
C7—H7A	0.9800	C14—H14B	0.9800
C7—H7B	0.9800	C14—H14C	0.9800
C7—H7C	0.9800	C15—H15A	0.9800
C8—H8A	0.9800	C15—H15B	0.9800
C8—H8B	0.9800	C15—H15C	0.9800
C8—H8C	0.9800	C16—H16A	0.9800
C9—H9A	0.9800	C16—H16B	0.9800
C9—H9B	0.9800	C16—H16C	0.9800
C9—H9C	0.9800	O3—H31	0.78 (3)
N6A—C10	1.333 (3)	O3—H32	0.86 (4)
N6A—C12	1.475 (3)	O4—H41	0.84 (3)
N6A—C11	1.475 (3)	O4—H42	0.83 (4)
N7A—C10	1.336 (3)	O5—H51	0.85 (3)
N7A—C15	1.464 (3)	O5—H52	0.74 (4)
N7A—C16	1.480 (3)		
			
N1—C1—O1	179.2 (3)	C10—N8A—C14	121.18 (18)
N2—C2—O2	179.1 (2)	C13—N8A—C14	116.95 (18)
N4—C3—N3	121.01 (16)	C10—N6B—C15	114.4 (7)
N4—C3—N5	119.77 (17)	C10—N6B—C14	110.0 (7)
N3—C3—N5	119.21 (17)	C15—N6B—C14	134.5 (7)
C3—N3—C7	122.40 (16)	C10—N7B—C11	113.4 (7)
C3—N3—C6	121.41 (16)	C10—N7B—C16	111.5 (7)
C7—N3—C6	116.15 (16)	C11—N7B—C16	135.0 (7)
C3—N4—C8	121.87 (17)	C10—N8B—C13	118.2 (7)
C3—N4—C9	122.10 (16)	C10—N8B—C12	110.3 (6)
C8—N4—C9	116.01 (16)	C13—N8B—C12	131.3 (6)
C3—N5—C5	121.89 (17)	N6A—C10—N7A	119.49 (18)
C3—N5—C4	121.58 (18)	N6A—C10—N8A	119.80 (18)
C5—N5—C4	116.52 (17)	N7A—C10—N8A	120.72 (18)
N5—C4—H4A	109.5	N8B—C10—N6B	120.0 (6)
N5—C4—H4B	109.5	N8B—C10—N7B	121.8 (7)
H4A—C4—H4B	109.5	N6B—C10—N7B	118.0 (7)
N5—C4—H4C	109.5	N6A—C11—H11A	109.5
H4A—C4—H4C	109.5	N6A—C11—H11B	109.5
H4B—C4—H4C	109.5	H11A—C11—H11B	109.5
N5—C5—H5A	109.5	N6A—C11—H11C	109.5
N5—C5—H5B	109.5	H11A—C11—H11C	109.5
H5A—C5—H5B	109.5	H11B—C11—H11C	109.5
N5—C5—H5C	109.5	N6A—C12—H12A	109.5
H5A—C5—H5C	109.5	N6A—C12—H12B	109.5
H5B—C5—H5C	109.5	H12A—C12—H12B	109.5
N3—C6—H6A	109.5	N6A—C12—H12C	109.5
N3—C6—H6B	109.5	H12A—C12—H12C	109.5
H6A—C6—H6B	109.5	H12B—C12—H12C	109.5
N3—C6—H6C	109.5	N8A—C13—H13A	109.5
H6A—C6—H6C	109.5	N8A—C13—H13B	109.5
H6B—C6—H6C	109.5	H13A—C13—H13B	109.5
N3—C7—H7A	109.5	N8A—C13—H13C	109.5
N3—C7—H7B	109.5	H13A—C13—H13C	109.5
H7A—C7—H7B	109.5	H13B—C13—H13C	109.5
N3—C7—H7C	109.5	N8A—C14—H14A	109.5
H7A—C7—H7C	109.5	N8A—C14—H14B	109.5
H7B—C7—H7C	109.5	H14A—C14—H14B	109.5
N4—C8—H8A	109.5	N8A—C14—H14C	109.5
N4—C8—H8B	109.5	H14A—C14—H14C	109.5
H8A—C8—H8B	109.5	H14B—C14—H14C	109.5
N4—C8—H8C	109.5	N7A—C15—H15A	109.5
H8A—C8—H8C	109.5	N7A—C15—H15B	109.5
H8B—C8—H8C	109.5	H15A—C15—H15B	109.5
N4—C9—H9A	109.5	N7A—C15—H15C	109.5
N4—C9—H9B	109.5	H15A—C15—H15C	109.5
H9A—C9—H9B	109.5	H15B—C15—H15C	109.5
N4—C9—H9C	109.5	N7A—C16—H16A	109.5
H9A—C9—H9C	109.5	N7A—C16—H16B	109.5
H9B—C9—H9C	109.5	H16A—C16—H16B	109.5
C10—N6A—C12	120.70 (18)	N7A—C16—H16C	109.5
C10—N6A—C11	121.16 (18)	H16A—C16—H16C	109.5
C12—N6A—C11	118.14 (19)	H16B—C16—H16C	109.5
C10—N7A—C15	121.83 (18)	H31—O3—H32	106 (3)
C10—N7A—C16	120.70 (19)	H41—O4—H42	102 (3)
C15—N7A—C16	117.45 (18)	H51—O5—H52	105 (3)
C10—N8A—C13	121.86 (18)		
			
N4—C3—N3—C7	−146.45 (19)	C12—N6A—C10—N8A	34.3 (3)
N5—C3—N3—C7	32.4 (3)	C11—N6A—C10—N8A	−146.11 (19)
N4—C3—N3—C6	31.5 (3)	C15—N7A—C10—N6A	−147.2 (2)
N5—C3—N3—C6	−149.64 (18)	C16—N7A—C10—N6A	34.4 (3)
N3—C3—N4—C8	30.4 (3)	C15—N7A—C10—N8A	32.5 (3)
N5—C3—N4—C8	−148.39 (19)	C16—N7A—C10—N8A	−145.9 (2)
N3—C3—N4—C9	−147.87 (18)	C13—N8A—C10—N6A	31.7 (3)
N5—C3—N4—C9	33.3 (3)	C14—N8A—C10—N6A	−147.48 (19)
N4—C3—N5—C5	−145.34 (19)	C13—N8A—C10—N7A	−148.04 (19)
N3—C3—N5—C5	35.8 (3)	C14—N8A—C10—N7A	32.8 (3)
N4—C3—N5—C4	33.1 (3)	C15—N6B—C10—N8B	150.1 (7)
N3—C3—N5—C4	−145.71 (19)	C14—N6B—C10—N8B	−19.7 (10)
C13—N8B—C10—N6B	−34.6 (10)	C15—N6B—C10—N7B	−35.3 (10)
C12—N8B—C10—N6B	150.7 (6)	C14—N6B—C10—N7B	154.9 (6)
C13—N8B—C10—N7B	151.0 (7)	C11—N7B—C10—N8B	−32.9 (10)
C12—N8B—C10—N7B	−23.7 (9)	C16—N7B—C10—N8B	150.9 (7)
C12—N6A—C10—N7A	−146.0 (2)	C11—N7B—C10—N6B	152.6 (7)
C11—N6A—C10—N7A	33.6 (3)	C16—N7B—C10—N6B	−23.6 (10)

### Hydrogen-bond geometry (Å, º)

**Table:**

*D*—H···*A*	*D*—H	H···*A*	*D*···*A*	*D*—H···*A*
O3—H31···N2	0.78 (3)	2.00 (3)	2.780 (3)	176 (3)
O3—H32···O5^i^	0.86 (4)	2.00 (4)	2.858 (4)	172 (3)
O4—H42···O3^ii^	0.83 (4)	2.04 (4)	2.852 (4)	164 (3)
O4—H41···N1^ii^	0.84 (3)	2.00 (3)	2.833 (3)	173 (3)
O5—H51···O2^iii^	0.85 (3)	1.92 (3)	2.761 (3)	175 (3)
O5—H52···O1^iv^	0.74 (4)	2.10 (4)	2.840 (4)	177 (3)

Symmetry codes: (i) *x*−1, −*y*, *z*−1/2; (ii) *x*+1, *y*, *z*; (iii) *x*, −*y*, *z*+1/2; (iv) *x*+1/2, −*y*+1/2, *z*+1/2.
