# Supplementary figures and images for: Crystal structure of N,N,N′,N′,N′′,N′′-hexa­methyl­guanidinium cyanate 1.5-hydrate
Source: Acta Crystallogr E Crystallogr Commun. 2015 Dec 24;71(Pt 12):o1076–7. doi: 10.1107/S2056989015024317 (PMC4719987; doi:10.1107/S2056989015024317)

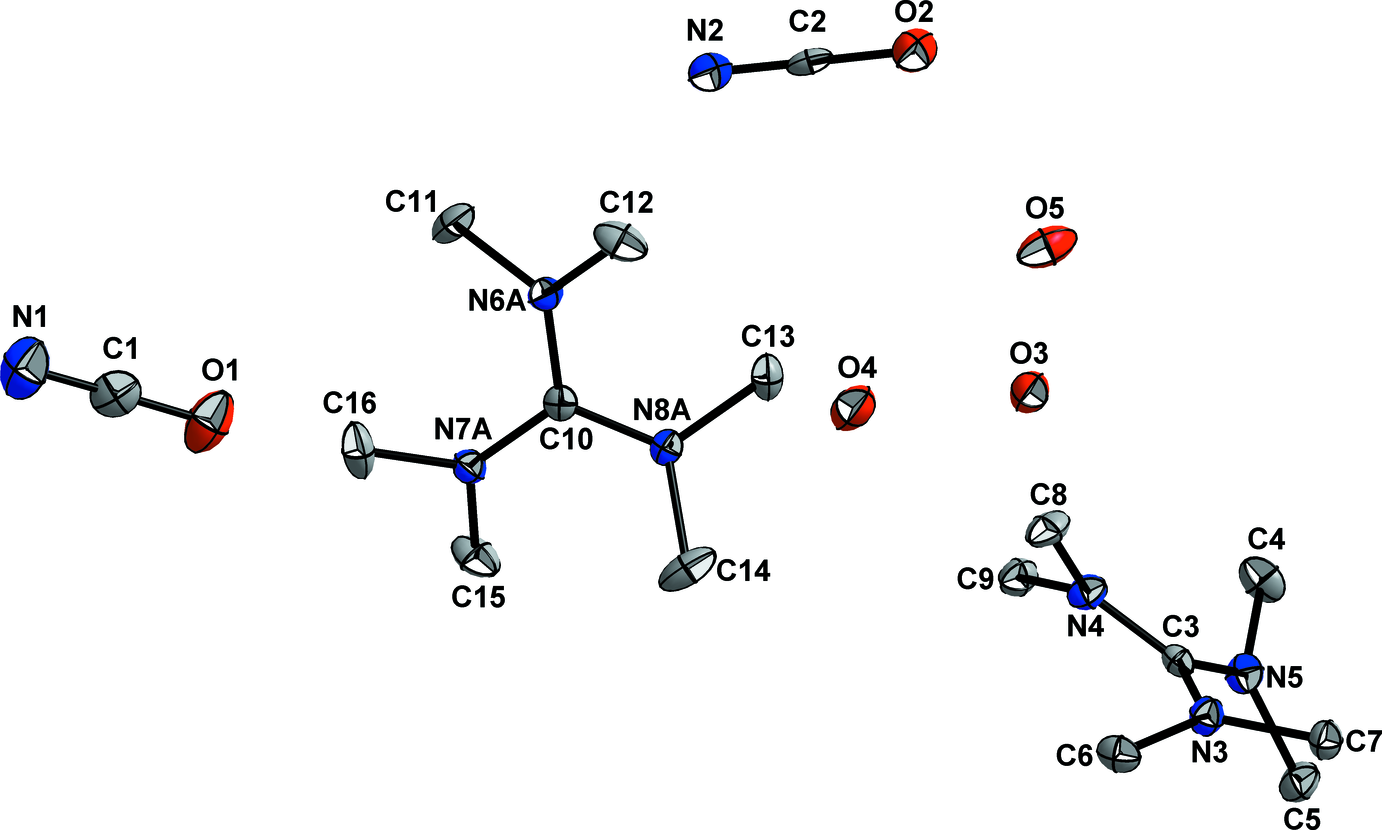

Supplement: Supplementary file 3 [file e-71-o1076-fig1.tif]

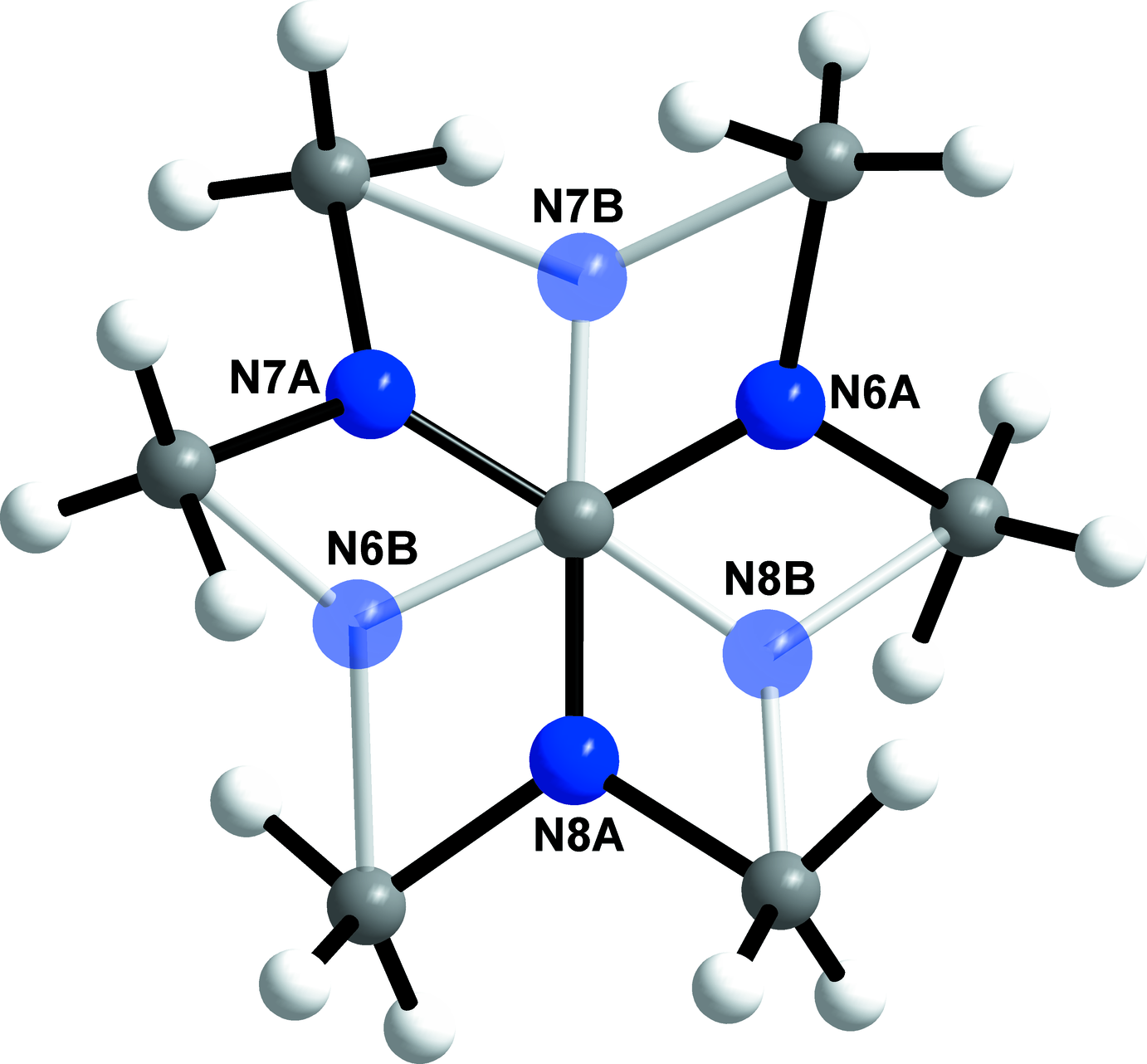

Supplement: Supplementary file 4 [file e-71-o1076-fig2.tif]

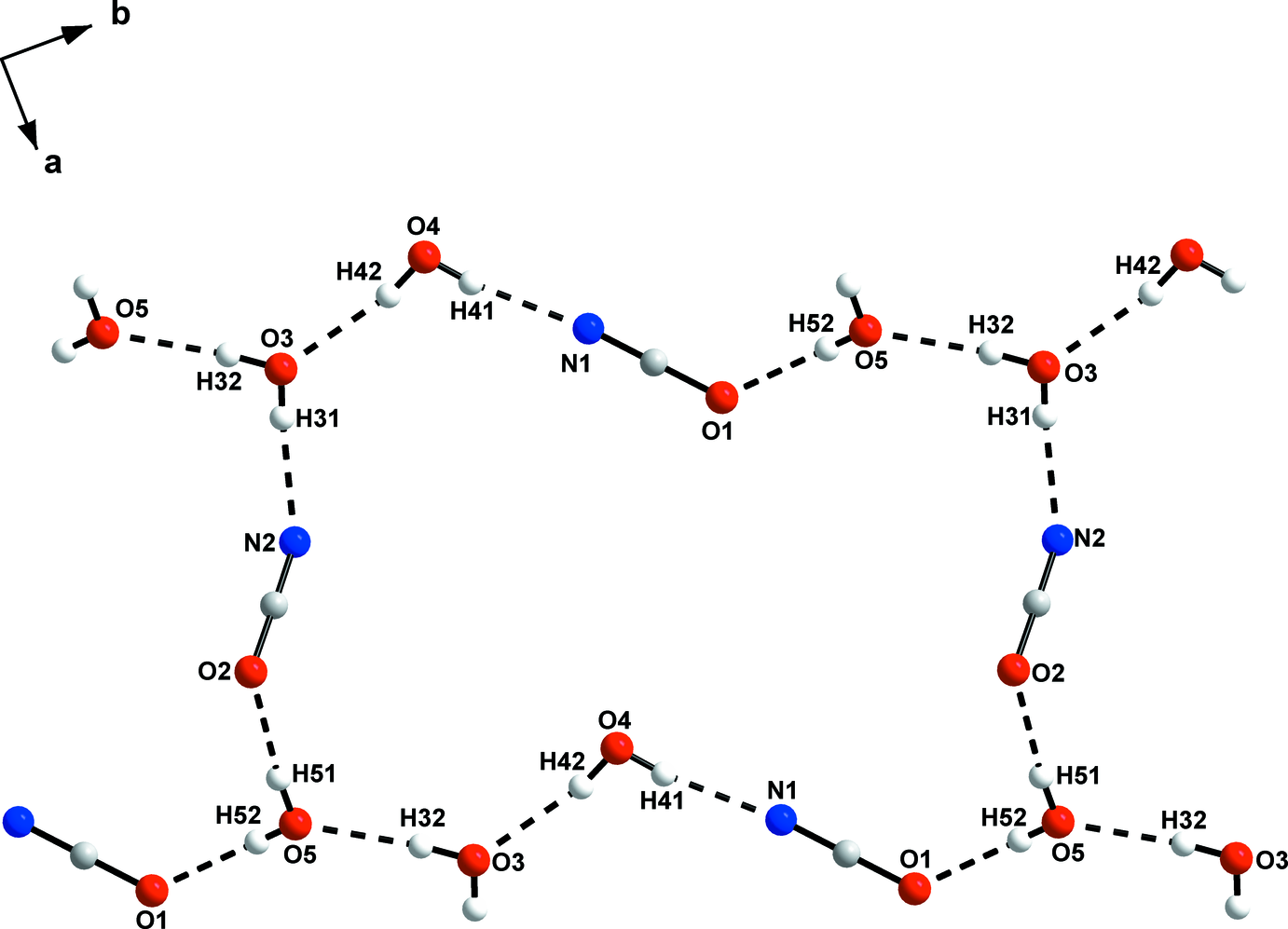

Supplement: Supplementary file 5 [file e-71-o1076-fig3.tif]

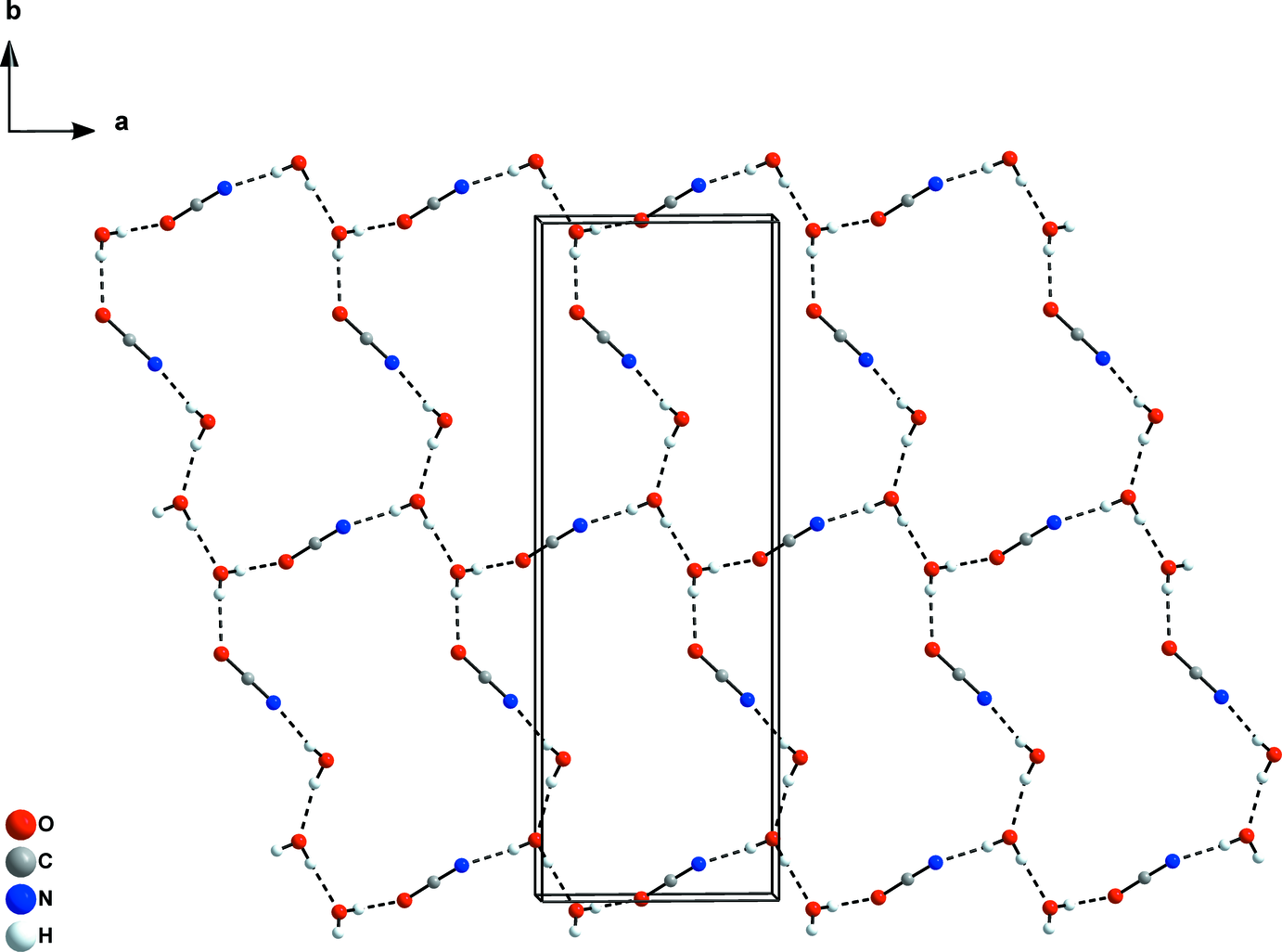

Supplement: Supplementary file 6 [file e-71-o1076-fig4.tif]

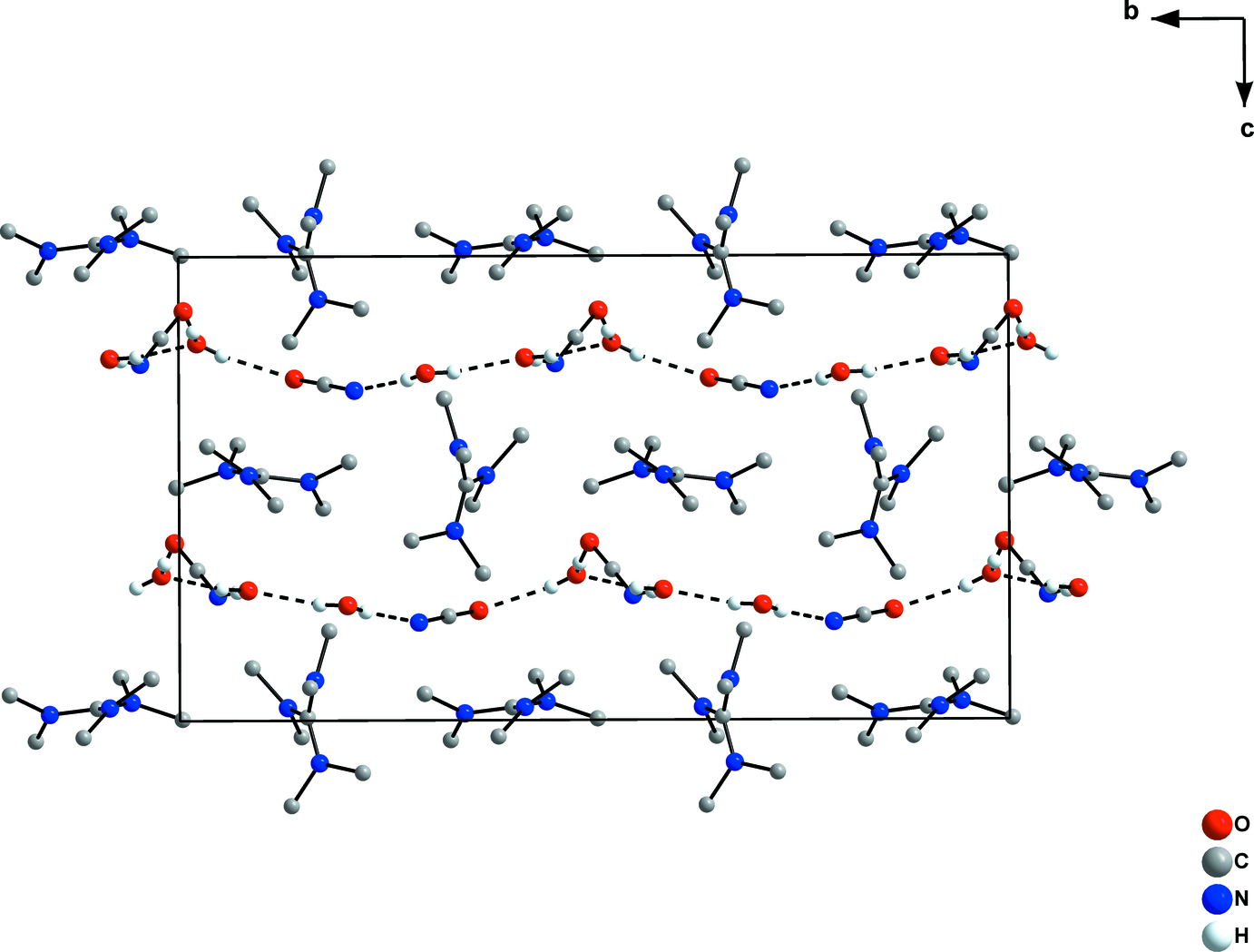

Supplement: Supplementary file 7 [file e-71-o1076-fig5.tif]
